# Perspectives of TGF-β inhibition in pancreatic and hepatocellular carcinomas

**DOI:** 10.18632/oncotarget.1569

**Published:** 2013-12-18

**Authors:** Cindy Neuzillet, Armand de Gramont, Annemilaï Tijeras-Raballand, Louis de Mestier, Jérome Cros, Sandrine Faivre, Eric Raymond

**Affiliations:** ^1^ INSERM U728 & U773 and Department of Medical Oncology, Beaujon University Hospital (AP-HP – PRES Paris 7 Diderot), 100 boulevard du Général Leclerc, Clichy, France; ^2^ AAREC Filia Research, 1 place Paul Verlaine, Boulogne-Billancourt, France; ^3^ Department of Pathology, Beaujon University Hospital (AP-HP – PRES Paris 7 Diderot), 100 boulevard du Général Leclerc, Clichy, France

**Keywords:** SMAD, stellate cells, extracellular matrix, EMT, TGF-β inhibitors

## Abstract

Advanced pancreatic ductal adenocarcinoma (PDAC) and hepatocellular carcinoma (HCC) are non-curable diseases with a particularly poor prognosis. Over the last decade, research has increasingly focused on the microenvironment surrounding cancer cells, and its role in tumour development and progression. PDAC and HCC differ markedly regarding their pathological features: PDAC are typically stromal-predominant, desmoplastic, poorly vascularized tumours, whereas HCC are cellular and highly vascularized. Despite these very different settings, PDAC and HCC share transforming growth factor-β (TGF-β) as a common key-signalling mediator, involved in epithelial-to-mesenchymal transition, invasion, and stroma-tumour dialogue. Recently, novel drugs blocking the TGF-β pathway have entered clinical evaluation demonstrating activity in patients with advanced PDAC and HCC. TGF-β signalling is complex and mediates both pro- and anti-tumoural activities in cancer cells depending on their context, in space and time, and their microenvironment. In this review we provide a comprehensive overview of the role of the TGF-β pathway and its deregulation in PDAC and HCC development and progression at the cellular and microenvironment levels. We also summarize key preclinical and clinical data on the role of TGF-β as a target for therapeutic intervention in PDAC and HCC, and explore perspectives to optimize TGF-β inhibition therapy

## INTRODUCTION

Advanced pancreatic ductal adenocarcinoma (PDAC) and hepatocellular carcinoma (HCC) have remarkably poor prognosis. Synchronous metastases are identified in 50% of PDAC patients at diagnosis[1] and preclinical models suggest that metastatic dissemination, the leading cause of PDAC-related death, might exist even before the primary tumour is detectable[2]. Unlike PDAC, HCCs are mostly locoregional-spreading tumours, with extra-hepatic metastases being a late event. Mortality is closely related to liver dysfunction or portal hypertension complications due to underlying liver disease, portal thrombosis and/or massive tumour involvement[3]. Treatment options are limited for both malignancies with only a minority of PDAC and HCC patients being candidates for surgery due to disease extent and/or liver dysfunction. Advanced PDAC is a contender for cytotoxic-based therapies (gemcitabine, nab-paclitaxel, or combined 5-FU/irinotecan/oxaliplatin as the FOLFIRINOX regimen), while sorafenib, an oral multi-tyrosine kinase inhibitor targeting the VEGFR, PDGFR and Raf pathways is the only approved systemic therapy for advanced HCC patients[4, 5]. Both PDAC and HCC are clearly therapeutically challenging digestive cancers and new therapeutic options are urgently needed.

Over the last decade, research has increasingly focused on the microenvironment surrounding cancer cells, and its role in tumour development and progression. PDAC and HCC differ markedly regarding their pathological features: PDAC are typically stromal-predominant, desmoplastic, poorly vascularized tumours, whereas HCC are cellular and highly vascularized[1, 6]. Despite these contrasting microenvironment settings, PDAC and HCC share transforming growth factor-β (TGF-β) as a common key signalling mediator. TGF-β is involved in epithelial-to-mesenchymal transition (EMT), invasion, and stroma-tumour dialogue in both tumour types.

In the first part of this review, we provide a comprehensive overview of the roles played by the TGF-β pathway and its deregulation in PDAC and HCC development and progression, at the cellular and microenvironment levels. We then go on to summarize key preclinical and clinical data describing the role of TGF-β as a target for therapeutic intervention in PDAC and HCC, and explore perspectives to optimize TGF-β inhibition therapy.

## 2 ROLE OF TGF-β AT THE CELLULAR LEVEL

### 2.1 TGF-β pathway in a nutshell

**Figure 1:**
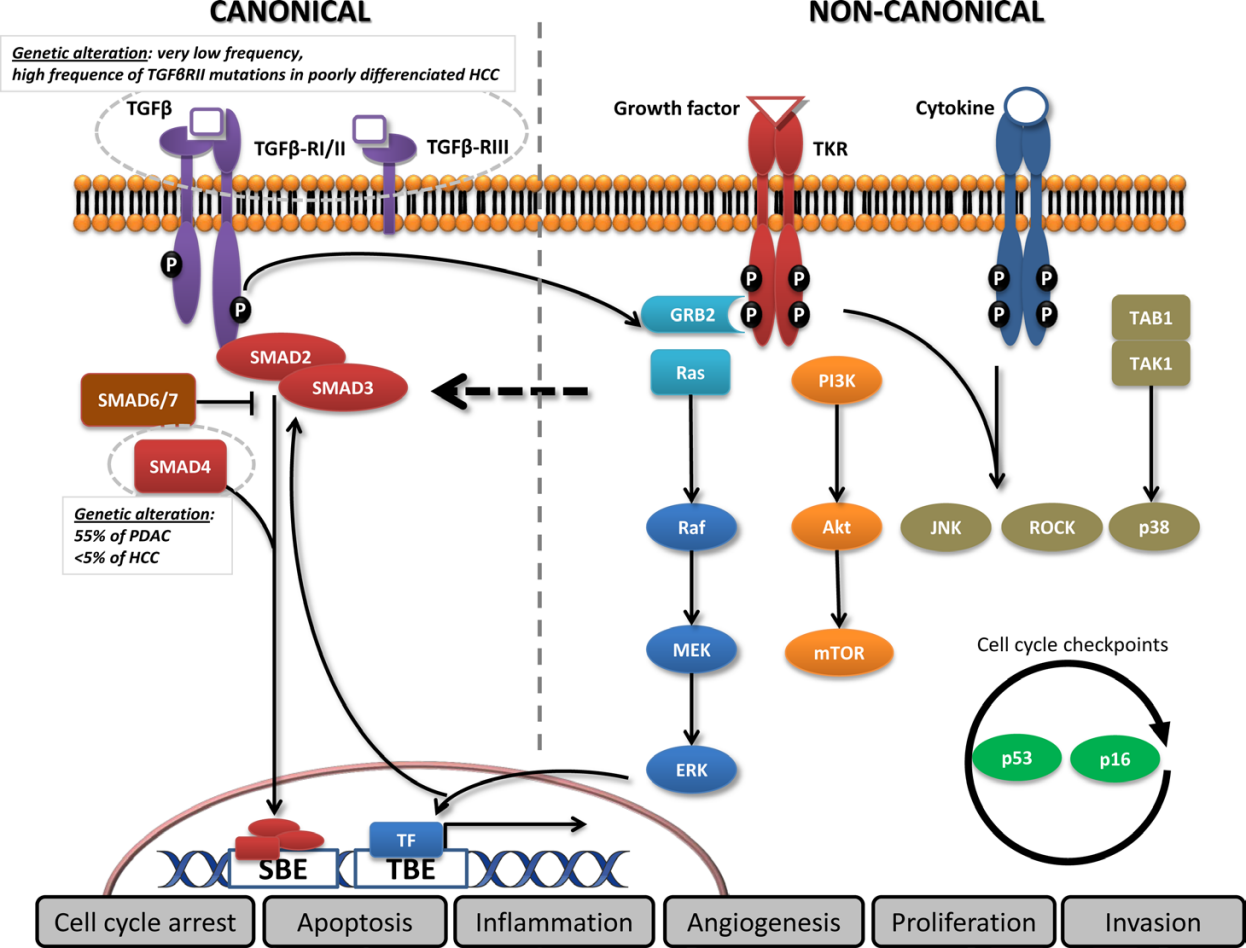
Canonical and non-canonical TGF-β pathways In the canonical pathway, the three TGF-β ligand isoforms, TGF-β1, TGF-β2, and TGF-β3, are synthesized as precursors and bind to form the latent TGF-β complex before being secreted[138]. After extracellular activation, TGF-β ligands bind to the membranous TGF-β type III receptor or the TGF-β type II receptor (TGF-βRII) homodimers with high affinity. TGF-βRII binding allows dimerization with TGF-β type I receptor (TGF-βRI) homodimers, activation of the TGF-βRI kinase domain and signal transduction via phosphorylation of the C-terminus of receptor-regulated SMADs (R-SMAD), SMAD2 and SMAD3. The TGF-βR dimer then forms a heterotrimeric complex with SMAD4 which translocates and accumulates in the nucleus[139, 140]. TGF-β dependent signalling can activate or repress hundreds of target genes through the interaction of SMADs with various transcription factors (TF). SMAD activities are regulated through several mechanisms: SMAD2/3 nucleocytoplasmic shuttling, binding to anchor proteins such as SARA, phosphorylation (e.g., by ERK, JNK, and p38 MAPK), Smurf (SMAD-ubiquitination-regulatory factor)-dependent degradation, or via expression of inhibitory SMAD6 and SMAD7[141]. In the non-canonical pathway, TGF-β signalling activates SMAD-independent pathways such as PI3K/AKT, MAPK pathways (ERK, JNK, and p38 MAPK) as well as NF-κB, Rho/Rac1, Cdc42, FAK, Src, Abl[142]. Moreover, transversal signalling, especially at the SMAD level, allows TGF-β pathway activation to integrate signals from integrins, Notch, Wnt, TNF-α, or EGF-dependent pathways as well as signals from cellular processes such as the cell cycle or apoptosis machineries[143]. The TGF-β signalling pathway thus has pleiotropic functions regulating cell growth, differentiation, apoptosis, cell motility, extracellular matrix production, angiogenesis and cellular immune response[144].

TGF-β is a well-recognised actor of development and is involved in the regulation of cell proliferation, differentiation, invasion, and inflammation. Key features of the TGF-β signalling pathway are depicted in figure 1. Deletion of the TGFβ1 or TGFβRII gene in mice resulted in defects in haematopoiesis, vasculogenesis, and endothelial differentiation of extra-embryonic tissues, while knockout mice for SMAD2 or SMAD4 displayed abnormal mesoderm formation[7]. Mice knockout for TGFβ1, TGFβRII, or SMAD4 genes are more likely to have spontaneous tumour development and excessive inflammatory responses, confirming the tumour suppressor properties of the TGF-β pathway[7]. In humans, mutations in the TGFβRII gene have been associated with multiple syndromes, and SMAD4 mutation is genetically responsible for familial juvenile polyposis, an autosomal dominant disease characterized by predisposition to gastrointestinal polyps and cancers.

Hijacking crucial biological functions by deregulating the TGF-β signalling pathway has recently emerged as a leading area of preclinical and clinical cancer research. The TGF-β pathway has both pro- and anti-tumoural activities[8-10]. On one hand, the TGF-β pathway promotes cell cycle arrest, apoptosis, and autophagy in epithelial cells, and also inhibits infammation[8-10]. On the other hand, by promoting angiogenesis, cell motility, invasion, EMT, or cell stemness, the TGF-β pathway promotes tumour progression[8-10]. The current paradigm for the role of TGF-β in carcinogenesis is that accumulation of genetic alterations in the TGF-β pathway drives the pathway's evolution from tumour-suppressive to tumour-promoting functions[9]. However, as we discuss in this review, it is critical not to isolate the role of TGF-β in tumour cells from their context in space and time, and from their microenvironment.

### 2.2 TGF-β pathway alterations in tumour cells

Alterations in TGF-β signalling are common in cancer. The TGF-β pathway is one of the 12 central cellular signalling pathways and processes that are frequently genetically altered in PDAC[11]. Regarding the TGF-β ligand, TGF-β mutations are very uncommon and to date none have been reported in PDAC or HCC [COSMIC Database]. TGF-β1 expression is detected immunohistochemically in about 40% of PDACs, and high TGF-β1 plasma levels have been measured in HCC patients compared to patients with cirrhosis only, with decreasing levels in patients who underwent effective HCC therapy[12-15]. Several studies have evaluated the prognostic significance of TGF-β levels in both PDAC and HCC[15-23]. However, published results are conflicting and no real consensus currently exists. For TGF-β receptors, TGFβRI gene alterations are uncommon (< 2%), whereas mutations in the TGFβRII gene are present at high frequencies in some cancers including HCC. Higher rates are found in poorly differentiated HCC tumours (53% of cases), but mutations are infrequent in PDAC (4%) [COSMIC Database][24, 25]. Low levels of TGF-βRI, TGF-βRII and TGF-βRIII have been observed and may be associated with poor prognosis in various cancers[26]. However, similar to TGF-β ligand levels, analyses of their prognostic value are conflicting and no consensus has been reached[27].

Downstream of the TGF-β receptors, mutations in SMAD2 and SMAD3 genes are infrequent and reports on the prognostic value of their expression level in PDAC and HCC are sparse [COSMIC Database]. In patients with hepatitis B and hepatitis C virus chronic infection, the phospho-SMAD2/3 L isoform was associated with a higher risk of developing HCC[28, 29]. SMAD4 inactivation is one of the most common alterations in PDAC (50-60%), caused by deletion, mutation or epigenetic modification, and many studies have shown that loss of SMAD4 expression is associated with a worse prognosis[30-32]. For instance, in a genetic analysis of 39 frequent mutations in 89 patients with resected PDAC, SMAD4 mutations were significantly associated with shorter survival[33]. In the largest study to date by Bachet et al.[34], loss of SMAD4 expression had no prognostic value but was predictive of adjuvant gemcitabine benefit. These apparently conflicting results may arise from the different techniques used to evaluate SMAD4 alterations and differences in patient populations and treatments. In HCC, SMAD4 mutations are uncommon (> 2%) [COSMIC Database]. However, there are also reports of reduced SMAD4 expression in HCC cells compared to surrounding liver tissue[35]. In contrast, increased expression was reported in subsets of patients with chronic viral hepatitis infection or in association with increased TGF-βRII overexpression, and was linked with poor prognosis[35-37]. Emerging data regarding inhibitory SMADs (I-SMAD) showed that low levels of SMAD7 correlated with increased recurrence and shorter survival in PDAC and HCC patients[38, 39].

### 2.3 TGF-β pathway in early events of carcinogenesis

The role of the TGF-β pathway in carcinogenesis is currently controversial. In its primary function, TGF-β has major tumour-suppressive properties, suggesting that TGF-β pathway inactivation is mandatory for tumour cell growth[9, 10]. In PDAC, mutation-driven KRAS over-activation is a very early alteration present in almost all tumours. Isolated expression of activated KRAS in mice drives the formation of pancreatic intraepithelial neoplasia (PanIN) and PDAC, however lesion progression is slow, and TGF-β pathway activation is sustained[40, 41]. SMAD4 or TGFβRII deletion in KRASG12D transgenic mice dramatically increased tumour aggressiveness, with accelerated PanIN and PDAC development, suggesting cooperation between these genetic alterations and mutant KRAS[42-44]. The importance of SMAD4 loss-of-function in PDAC carcinogenesis is supported by the fact that SMAD4 inactivation is observed in most tumours. In the context of tumour progression, SMAD4 loss-of-function may not only be necessary for counteracting TGF-β anti-proliferative effects, but may also contribute to rewiring the cells' processing system, translating TGF-β input signals into different outputs. In in vitro and in vivo experiments, adding TGF-β to SMAD4-null cell lines resulted in increased proliferation rather than tumour suppression[45].

In HCC, the low frequency of SMAD4 inactivation implies that different mechanisms are engaged in eliciting TGF-β inhibitory functions. The current paradigm, supported by studies involving expression of TGF-β pathway components in patient tissues, is that early carcinogenesis relies on low TGF-β pathway activity or “early TGF-β signature”[46, 47]. Early TGF-β pathway attenuation is characterized by activation of a negative feedback loop through increased expression of the I-SMAD SMAD7, and expression of two negative regulators of the TGF-β pathway, the SKI-like and TGF-β-induced factor genes, which are both co-repressors of the SMAD2/3-dependent transcription complex[46]. This early TGF-β signature is also characterized by expression of the DNA damage gene family Gadd45, which is involved in cell cycle arrest and apoptosis[46, 48]. Given this strong induction of anti-tumourigenic genes, early tumour promoting activity of TGF-β requires a cellular context with imbalanced sensitivity towards pro- and anti-growth signals. For example, p16INK4 gene alterations are present in up to 90% of HCCs. They favour insensitivity to anti-growth signals by affecting both the cell cycle through relieving cyclin D/CDK4,6 complex inhibition, and the apoptotic machinery by lowering p53 activation[32]. Similarly, over-activation of pro-mitogenic pathways such as EGFR, or TGF-β-dependent cytokine expression (EGF, PDGF, IGF-1, HGF, FGF, etc.) may modify TGF-β response. For example, the Ras-ERK pathway can transduce signals downstream of TGF-βR, and ERK may modify SMAD-dependent signalling by modulating SMAD2/3 phosphorylation. SMADs are differentially phosphorylated by TGF-βR (C-terminal region) and ERK (linker L region), resulting in various phosphoisoforms (C, L, L/C) with distinct localization and cellular effects[49]. Hyperactivation of the PI3K/AKT pathway also cooperates with the TGF-β pathway in hepatocarcinogenesis in cirrhotic livers[50].

To summarize, in early steps of carcinogenesis, TGF-β displays tumour-suppressive properties, with mechanistic differences between PDAC and HCC models. In PDAC, the TGF-β pathway initially drives anti-proliferative signals until SMAD4 silencing occurs, modifying the outputs of TGF-β pathway activation. In HCC, the anti-proliferative effects of TGF-β are bypassed via mitogenic signals or impaired sensitivity to anti-growth signals.

### 2.4 TGF-β pathway in promoting metastasis

Most tumours acquire a metastatic phenotype during progression, developing the capacity to invade surrounding tissues, migrate and grow at distant sites via an EMT-dependent process. This process is linked to dedifferentiation, and epithelial cells undergo a phenotypic shift from having tight cell-cell junctions, clear basal and apical polarity, sheet-like growth architecture, with expression of epithelial markers such as E-cadherin, into spindle-like fusiform, motile cells expressing mesenchymal markers such as vimentin and N-cadherin. This change in morphology and remodelling of the extracellular matrix (ECM), notably through the expression of matrix metalloproteinases (MMPs), confers them with invading potential. Accumulating evidence shows that TGF-β has late-stage tumour effects particularly in promoting EMT and, as a consequence, cancer dissemination[9, 10].

Coulouarn et al.[46] showed that high TGF-β pathway activity with the “late TGF-β signature” favours late tumourigenic evolution in HCC, notably in terms of metastatic spreading. It is characterized by modulation of genes involved in cytoskeleton organization (e.g., vimentin and supervillin), cell adhesion (e.g., integrin-α6 and activated leucocyte cell adhesion molecule), and matrix remodelling and migration, along with expression of connective tissue growth factor (CTGF) and Rhob[46, 48, 51]. Interestingly, this late TGF-β signature is also characterized by the expression of the SNAI1 gene which encodes Snail, an E-cadherin transcription repressor[46, 48]. In addition, TGF-β is a major actor in EMT in PDAC, and cooperates with the activated Ras-ERK pathway to activate EMT transcription factors such as Snail and ZEB[32, 52].

Moreover, in both PDAC and HCC, TGF-β promotes tumour invasiveness through MMP induction. For example, MMP-2, MMP-9, MT-MMP1 and urokinase-like plasminogen activator are up-regulated in vitro by TGF-β1[53-55]. TGF-β1 inhibition reduces MMP production and cell invasiveness, possibly via partial EMT reversion, inhibition of integrin signalling, and reduced CTGF production[51, 56, 57].

In both PDAC and HCC, mesenchymal differentiation of tumour cells has been associated with poor prognosis, while TGF-β expression or TGF-β pathway activation correlates with the EMT status of tumour cells. In a recent analysis of a series of resected HCC specimens, a mesenchymal phenotype (high vimentin and low E-cadherin expression) was associated with shorter survival and enhanced TGF-β pathway activity (increased TGF-β1, phospho-SMAD-2, and phospho-β1 integrin expression)[58].

Crosstalk and TGF-β coupling with other signalling pathways may be critically important for tumour progression. As essential components of cell-to-matrix adhesion, integrins are involved in modulating the TGF-β response. In PDAC, αVβ6 integrin cooperates with TGF-β in its tumour-suppressor function whereas in HCC, αVβ1 integrins or α3β1 integrins are stimulated by TGF-β to promote tumour invasion[16, 56, 59]. TGF-β can activate and cooperate with multiple other pathways involved in invasion and metastasis (e.g., MAPK, PI3K/AKT/mTOR, NF-κB, Notch, Wnt, and CXCR4) through SMAD2/3-dependent and -independent signalling mechanisms[60]. For instance, in HCC, co-activation of the Wnt and TGF-β pathways define an HCC subclass with a more aggressive phenotype, and in PDAC SMAD4 was required to transduce Wnt signalling in a SMAD2/3-independent manner[61-63]. In contrast to early carcinogenesis, the presence of SMAD4 seems to be important for the metastatic potential of PDAC tumour cells in vivo[40]. Activation of the Ras-ERK and TGF-β-SMAD4 pathways was required for EMT induction and maintenance[52]. Mutated K-Ras and TGF-β cooperate to produce L/C forms of phospho-SMAD2 and phospho-SMAD3, which are translocated into the nucleus and activate transcription of pro-proliferative (c-Myc) and pro-invasive (MMPs) genes[49].

To summarize, TGF-β plays an important role in both PDAC and HCC in late-stage tumour progression by promoting EMT, invasion, and, as a result, cancer metastasis, in cooperation with other pathways.

## 3 ROLE OF TGF-β AT THE MICROENVIRONMENT LEVEL

### 3.1 PDAC: stromal avascular microenvironment

PDAC displays the most prominent desmoplastic stromal reaction of all epithelial tumours, often greater than the epithelial component of the tumour itself[1, 64, 65]. Fibrotic focus (evidence of intratumoural fbroblast proliferation following focal necrosis) and stromal abundance and activity (evaluated by collagen deposition and α-smooth actin immunostaining) correlated with poorer survival in resected PDAC patients, suggesting a prognostic role for desmoplasia in PDAC[66-69]. This desmoplastic stroma is a complex structure composed of ECM proteins and various cell types including pancreatic stellate cells (PSCs), endothelial cells and pericytes, nerve cells, immune cells, and bone marrow-derived stem cells[68].

Activated PSCs are responsible for excess ECM production in PDAC, and TGF-β1 is a key signalling factor in this process[70]. Löhr et al.[71] demonstrated that TGF-β1 overexpression induced up-regulation of ECM proteins in vitro in co-culture experiments in TGF-β1-transfected PDAC cells and fibroblasts, and also when fibroblasts were grown in conditioned medium from TGF-β1-transfected PDAC cells. In vivo, TGF-β1-transfected PDAC cells induced a rich stroma after orthotopic transplantation into nude mice pancreas. Consistent with this, Bachem et al.[72] showed that PDAC cell lines stimulated PSC proliferation. Using specific neutralizing antibodies, they demonstrated that the increase in ECM protein production was mediated by TGF-β1 and FGF2, while PSC proliferation was likely mediated by PDGF. In addition, after subcutaneous injection of combined PDAC cells and PSCs into immunodeficient mice, tumours grew faster than with PDAC cells injected alone. On histologic examination, mixed PSCs-PDAC cells tumours displayed intense desmoplastic reaction, but also an increased number of cancer cells themselves[72]. Additional studies, using other physiological orthotopic and transgenic mice models, also demonstrated that human PSCs within the tumour stimulated fibrosis, local tumour growth, and importantly, promoted regional and distant metastasis[73-75]. Strikingly, PSCs were also detected in metastatic nodules in the liver in mice, suggesting that PSCs can migrate with PDAC cells to establish a potentially tumour-favourable microenvironment at distant sites[73, 76]. Taken together, these data highlight the crucial role of the interactions between cancer cells and PSCs in tumour progression in PDAC, via TGF-β1 and desmoplasia[77, 78].

PSCs not only create a fibrotic microenvironment, but also contribute to make it hypoxic. Although activated PSCs produce pro-angiogenic factors such as VEGF, they are dominantly anti-angiogenic, through (1) enhancing anti-angiogenic endostatin production by PDAC cells, (2) compressing vessels by the dense and fibrotic stroma and (3) high interstitial pressure, all of which result in low vascularization and tumour hypoxia[64, 79]. In addition, hypoxia stimulates pro-fibrogenic functions of PSCs (production of ECM components and CTGF), thus perpetuating a hypoxia-fbrosis vicious cycle[80-82]. Avascular hypoxic microenvironments promote survival of anaerobic cancer cells that are intrinsically resistant to hypoxia-induced apoptosis[83]. This may explain the failure of anti-angiogenic treatment in PDAC[64, 84]. Moreover, hypoxia may select for tumour cells with a more aggressive phenotype[85], a well described phenomenon in other tumour types[86, 87]. This may be due to hypoxia-induced activation of EMT and invasion pathways such as HGF/c-Met or CXCR4/CXCL12, through HIF-1α-dependent and independent mechanisms[88-93]. Hypoxia-induced modulations of tumour metabolism (glycolysis, glutaminolysis, lactate efflux) may additionally contribute to increased aggressiveness[93]. The TGF-β pathway cooperates with hypoxia in these processes[94]. In preclinical studies, TGF-β inhibition by various agents (LY2109761, SD-208, and trabedersen) reduced PDAC cells invasion in vitro and metastasis in vivo[95-97]. Consequently, PSCs and TGF-β1 in PDAC desmoplasia may contribute to create a hypoxic microenvironment exerting a selection pressure toward a more invasive cancer cell phenotype.

To summarize, TGF-β is a major pro-fibrotic factor in PDAC carcinogenesis. TGF-β therapeutic inhibition in PDAC might thus result in stromal depletion, vascularization enhancement with improved drug delivery, and an anti-metastatic effect. An anti-fibrogenic effect has yet to be well studied due to the lack of relevant preclinical models mimicking the stromal complexity of PDAC[64].

### 3.2 HCC: cellular highly vascularized microenvironment

The role of TGF-β signalling is quite different in the HCC microenvironment. In contrast to PDAC, HCCs are typically hypervascularized tumours with predominant arterial perfusion[98, 99]. Angiogenesis plays an important role in HCC development and growth as suggested by high circulating VEGF levels and pathological studies showing the development of unpaired arteries, increased histological microvessel density, and VEGF immunostaining on tissue biopsies[100-102]. Consistent with this, HCCs are responsive to intra-arterial embolization and anti-angiogenic agents such as sorafenib or sunitinib, suggesting angiogenic dependence[98].

TGF-β plays a pro-tumourigenic role in HCC mainly by promoting angiogenesis[103]. Ito el al.[104] showed that TGF-β plasma levels were positively correlated with tumour vascularity assessed by celiac angiography. TGF-β signalling can induce angiogenic factors such as VEGF and CTGF in epithelial cells and the fbroblasts that promote these epithelial cells[105]. Mazzocca et al.[106] demonstrated that the TGF-βRI inhibitor LY2109761 displayed anti-angiogenic activity by inhibiting VEGF secretion. Mechanistically, LY2109761 blocked paracrine crosstalk between HCC cells and endothelial cells involving SMAD2/3-mediated signalling, and consequently the formation of blood vessels. Interestingly, this anti-angiogenic effect was more effective than that of bevacizumab, a specific anti-VEGF monoclonal antibody.

In addition, TGF-β-mediated CTGF production is also involved in ECM deposition in HCC, despite that HCC stromal content is much less abundant than in PDAC. CTGF plays an important role in the crosstalk between HCC cells and HSCs (or cancer-associated fibroblasts, CAFs) to control stroma production[51]. TGF-β circulating levels increase in line with collagen deposition and the reduction of ECM degradation[107]. The Gianelli group[51] showed that HCC cell lines producing high levels of CTGF generated high stromogenic tumours, which was reversed by CTGF knock-down. Upon TGF-β1 stimulation, low-CTGF HCC cells formed tumours with a high stromal content and CTGF expression, which was inhibited by treatment with LY2109761. Blocking TGF-β signalling with LY2109761 inhibited CTGF synthesis and release from HCC cells (and CAFs) and reduced tumour stromal content by inhibiting CAF proliferation[51]. Inhibiting this pro-fibrogenic role of TGF-β may be of particular interest in forms of HCC exhibiting dense stroma such as fibrolamellar HCC, similar to PDAC.

Overall, TGF-β promotes HCC vascularization, with an important role of VEGF and CTGF as paracrine mediators. TGF-β inhibition may mainly have an anti-angiogenic role in HCC.

### 3.3 TGF-β in PDAC and HCC cancer microenvironment: immune system deregulation

Many lines of preclinical evidence suggest that TGF-β plays a crucial role in immune regulation[108, 109]. The immune system is responsible for the early detection and destruction of cancer cells. Some cancer cells become immunologically invisible by passive avoidance of immune surveillance (i.e., cancer cell “hiding”). Another mechanism for escaping immune surveillance is to actively secrete cytokines that “blind” the immune system to the presence of abnormal antigens at the cancer cell surface. TGF-β1 is the most potent immunosuppressor and plays a crucial role in this process. TGF-β1-null mice exhibit a phenotype of excessive inflammatory response and early death, with multifocal inflammatory disease in many tissues and massive tissue infiltration by lymphocytes and macrophages[110, 111]. Interestingly, marked immunosuppression is observed in patients with PDAC or HCC[112]. Tumour-associated TGF-β1 downregulates the host immune response via several mechanisms: it (1) drives the T-helper (Th) balance toward the Th2 immune phenotype via IL-10 as an intermediate; (2) directly inhibits anti-tumoural Th1-type responses and M1-type macrophages; (3) suppresses cytotoxic CD8+ T-lymphocytes, natural killer lymphocytes and dendritic cells functions; (4) generates CD4+CD25+ T-regulatory cell (T-regs) that suppress activity of other lymphocyte populations; (5) promotes M2-type macrophages with pro-tumoural activity mediated by secretion of immunosuppressive cytokines (e.g., IL-10, TGF-β), pro-angiogenic factors (e.g., VEGF, MMP-9, CXC chemokines), pro-inflammatory cytokines (e.g., IL-6, TNFα, IL-1), and tumour growth factors, and generates reactive oxygen species with genotoxic activity[109, 113-115].

**Figure 2:**
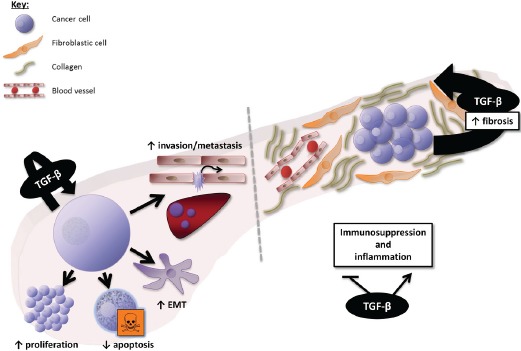
Overview of the effects of TGF-β signalling in PDAC At the cellular level, TGF-β induces proliferation and survival of PDAC cells in the late phase of PDAC carcinogenesis (after SMAD4 inactivation), and promotes epithelial-to-mesenchymal transition (EMT), invasion, and metastasis. At the microenvironment level, TGF-β is a key mediator of the dialogue between cancer and stellate cells (fibrotic cells), involved in the production of a dense fibrotic stroma and the resulting low vascularization of PDAC. TGF-β also deregulates the immune microenvironment toward immunosuppression and inappropriate inflammation.

**Figure 3:**
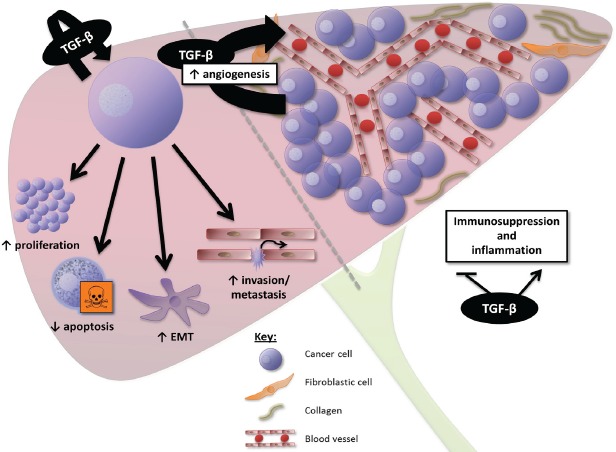
Overview of the effects of TGF-β signalling in HCC At the cellular level, TGF-β induces proliferation and survival of HCC cells displaying a “late TGF-β signature”, promoting epithelial-to-mesenchymal transition (EMT), invasion, and metastasis. At the microenvironment level, TGF-β is a key mediator of angiogenesis in HCC, contributing to the high vascularization of these tumours. TGF-β also generates a favourable immune microenvironment for tumour growth.

Together, these data suggest that TGF-β overexpression in PDAC and HCC generates a favourable immune microenvironment for tumour growth, and that TGF-β inhibition may contribute to restore anti-tumoural cytotoxic immune response. The effects of TGF-β signalling at the cellular and microenvironment levels in PDAC and HCC are summarized in figures 2 and 3, respectively.

## 4 TGF-β AT THE PATIENT LEVEL: TGF-β PATHWAY INHIBITORS IN THE CLINIC

Many TGF-β pathway inhibitors have been investigated in the preclinical setting, some of which are now in clinical development targeting either TGF-β ligands (TGF-β1, -β2, -β3) or receptors (TGF-βRI and TGF-βRII) (table 1). Nonetheless, very few of these inhibitors have been tested in the context of HCC or PDAC.

**Table 1:** TGF-β pathway inhibitors in development in hepatocellular and pancreatic carcinomas

Name	Targets	Trial identifier	Current Status
TGF-β ligand inhibitors			
Lerdelimumab Genzyme®	TGF-β2		Development stopped.
Metelimumab Genzyme®	TGF-β1		Development stopped.
Fresolimumab Genzyme®/Aventis®	TGF-β1, -β2, -β3		Not currently tested in PDAC or HCC. In progress in other cancer types.
LY2382770 Eli Lilly®	TGF-β1		Not currently tested in PDAC or HCC. In progress outside oncology.
Trabedersen Antisens Pharma®	TGF-β2	NCT00844064	Phase I/II completed. Phase II in progress. Results in a small PDAC cohort.
Lucanix NovaRx Corporation®	TGF-β2		Not currently tested in PDAC or HCC. In progress in other cancer types.
Disitertide Digna Biotech®	TGF-β1		Not currently tested in PDAC or HCC. In progress outside oncology.

TGF-β receptor inhibitors			
LY2157299 Eli Lilly®	TGF-βRI	NCT01246986 (HCC)	Phase I completed. Phase II in progress in both NCT01373164 (PDAC) PDAC and HCC. Early phase II results in HCC.
LY3022859 Eli Lilly®	TGF-βRII		Phase I in progress.

### 4.1 Inhibitors of TGF-β ligands

Four humanized monoclonal antibodies have been developed against TGF-β ligands, however data are not available in HCC and PDAC. Two antibodies, lerdelimumab directed against TGF-β2 and metelimumab directed against TGF-β1, were stopped for lack of efficacy before being evaluated in oncology. Fresolimumab, a pan-TGF-β antibody still under investigation, has completed phase II studies in glioma and relapsed malignant pleural mesothelioma, phase I in renal cell carcinoma and malignant melanoma, and is currently in phase I in metastatic breast cancer in association with radiotherapy. LY2382770, targeting TGF-β1, is currently being evaluated in diabetic kidney conditions but not in oncology.

Trabedersen (AP12009) is an antisense oligonucleotide targeting TGF-β2. In preclinical models, it displayed potent anti-tumour efficacy in TGF-β2-overexpressing PDAC cells, and drastically inhibited cell invasion, and also inhibited tumour growth, angiogenesis and lymph node metastasis in an orthotopic xenograft mouse model of metastatic PDAC[97]. It is currently in phase II development in glioblastoma. In a phase I/II study, a cohort of nine advanced PDAC patients received intravenous trabedersen (140 mg/m^2^/day, 4 days on/10 days off) as second-line therapy. Toxicities were limited and survival analysis showed a remarkable median overall survival of 13.4 months. One patient with liver metastasis had a complete response[116]. No clinical data are available in HCC to date.

Lucanix (belagenpumatucel-L) is a TGF-β2 antisense gene-modified allogeneic tumour cell vaccine that has completed phase II evaluation and is currently in a phase III study against placebo in non-small cell lung cancer (NSCLC) patients as maintenance therapy after front-line treatment[117]. Phase II results showed good tolerance and a survival rate of 47% in stage IIIB and IV NSCLC patients[1 17]. Lucanix has not yet been evaluated in PDAC or HCC.

Disitertide (P144) is a peptidic TGF-β1 inhibitor specifically designed to block the interaction with its receptor. In a mouse model of metastatic colorectal carcinoma, P144 inhibited tumour growth, liver metastasis, EMT and angiogenesis[118]. Phase I studies of topical application for skin fibrosis are completed but it is yet to be evaluated in oncology.

### 4.2 Inhibitors of TGF-β receptors

LY3022859 (IMC-TR1) is a monoclonal antibody against TGF-βRII that has just entered phase I clinical trial in patients with advanced solid tumors. No data are yet available in HCC or PDAAC

LY2157299 is a small molecule inhibitor of TGF-βRI. To date, it is the most advanced TGF-β signalling inhibitor under clinical development in HCC and PDAC. In preclinical models, LY2157299 has anti-tumour activity in NSCLC, breast and HCC models, affecting mainly tumour migration and invasion rather than proliferation 119]. In a triple-negative breast cancer xenograft model, TGF-β inhibition was synergistic with chemotherapy preventing the development of cancer stem cells[120]. Phase I evaluation showed a good safety profile and durable responses beyond one year in three patients. Several Phase II studies with LY2157299 are ongoing, including as second-line treatment in HCC after sorafenib (NCT01246986), in association with gemcitabine in advanced PDAC (NCT01373164), as well as in glioblastoma. Preliminary results of the HCC phase II were presented at the ASCO and ILCA 2013 meeting[121]; 106 patients have been randomized to receive LY2157299 at 160 or 300 mg/day. LY2157299 safety profile was suitable for patients with Child-Pugh A/B7 HCC. Median time to progression was 12 weeks. LY2157299 treatment was associated with AFP responses, reduction in TGF-β1 and E-cadherin levels, and time to tumor progression was increased in patients with AFP and TGF- β1 levels reduction from baseline. Further analysis is expected to confirm the signal to launch phase III clinical trials in patients with PDAC and/or HCC.

## 5 PERSPECTIVES AND CONCLUSIONS

The future of TGF-β-directed therapies is promising. However, many questions remain to be answered before optimal clinical use of these agents in PDAC and HCC is reached. At a fundamental level, there is a crucial need for pertinent and robust PDAC and HCC preclinical models to study the effects of the TGF-β pathway and its inhibition. Both these tumours have complex microenvironments in which cancer cells interact closely with ECM components and different cell types. Studying cancer cells in isolation in vitro is a far from optimal representation of in vivo circumstances. Compared to an in vivo setting, cells grown on two-dimensional (2D) tissue culture substrates differ markedly in their morphology, differentiation, and cell-cell and cell-matrix interactions[122, 123].

TGF-β effects can also differ considerably according to the features of the culture system used, e.g. its rigidity, composition, and structure[10]. Dedifferentiation was seen in hepatocytes grown on monolayers of dried stiff collagen, caused by a specific signalling network triggered by the ECM, activating focal adhesion kinase (FAK) via Src, which in turn activated Akt, causing resistance to TGF-β-induced apoptosis by antagonizing p38[124]. In contrast, FAK was not activated when hepatocytes were grown on a softer collagen gel, keeping them sensitive to TGF-β-induced apoptosis. In the PDAC COLO-357 cell line, Sempere et al.[125] showed that TGF-β had anti-proliferative effects on PDAC cells cultured on standard plastic plates or in soft agar, while it promoted cell growth in a three-dimensional (3D) culture system. These limits of classic 2D in vitro culture models may explain some of the discrepancies between in vitro and in vivo models evaluating TGF-β functioning.

Moreover, TGF-β is a paracrine-signalling molecule mediating interactions between cancer cells and stromal cells, including stellate cells. In vitro co-culture systems of cancer and stromal cells grown within or on top of reconstituted ECM gels are able to model cancer more realistically than 2D systems. These “organotypic cultures” were first described for human skin cancer and have been exploited to investigate various tumour types, including ovarian, breast, prostate and oesophageal, providing important insights into the role of frequently altered genes in biological behaviour and mechanisms of tumour invasion[126]. Such models may provide a more accurate prediction of the in vivo situation and may be useful to study the effects of TGF-β and TGF-β inhibition.

There are many clinical challenges to developing TGF-β inhibitors, notably patient selection, timing of treatment and predictive biomarkers. Given the dual effects of TGF-β on proliferation, TGF-β inhibition may only be beneficial in tumours expressing the “late TGF-β signature” (i.e. promoting proliferation and invasiveness)[9, 46]. More so than tumour cell characteristics, predictive power of biomarkers in terms of TGF-β inhibitor efficacy may be affected by the tumour microenvironment or a patient's overall blood biomarker profile. For example, patients with high intra-tumoural and/or circulating levels of TGF-β may be more likely to respond to specific TGF-β inhibitors. Thus, TGF-β inhibition (i.e., by inhibitors of TGF-β ligands) may be used to normalize tissue homeostasis by down-regulating excess TGF-β production of tumour and tumour-related tissues, with limited side effects on normal tissues. This raises the question of the timing and context in which TGF-β inhibition would be most beneficial. TGF-β inhibition may be of particular interest as a preventive strategy in HCC, in which TGF-β overproduction, as a driver of the fibrotic process of cirrhosis, precedes tumour formation and create a favourable microenvironment for tumour cells[9]. This may be useful both in the primary prevention setting and as adjuvant treatment after complete HCC ablation in cirrhotic patients. In addition, although there may be a potential hazard of stimulating synchronous occult tumours through the inhibition of TGF-β-induced tumour suppression (particularly with inhibitors of TGF-β receptors), early clinical results of TGF-β inhibition in HCC patients do not show evidence of malignant transformation from underlying cirrhotic livers[9, 121]. Nonetheless, in future multidisciplinary strategies, TGF-β inhibitors should be considered with caution after extensive liver resection, as the TGF-β pathway plays a crucial role during liver regeneration.

In contrast, in PDAC, TGF-β overproduction is more a consequence of tumour development, i.e. microenvironment remodelling by tumour cells. Thus, TGF-β levels are expected to decrease after tumour resection and a preventive or adjuvant role of TGF-β inhibition may be limited to the small fraction of resectable PDAC emerging in chronic pancreatitis. In this case, TGF-β inhibitors should be preferentially used in the advanced PDAC setting. Moreover, radiotherapy and chemotherapy induce TGF-β activity, possibly promoting metastatic progression, and high levels of TGF-β are associated with resistance to anticancer treatments[10, 127]. Then, combined TGF-β inhibition may enhance tumour sensitivity to chemotherapy and radiotherapy[10]. TGF-β inhibition should thus be tested in association with conventional cytotoxic chemotherapy, both in advanced PDAC and HCC.

As TGF-β inhibitors are mainly anti-invasive agents and can display dual effects on proliferation, clinical development of these agents raises the question of whether they should be used as monotherapy or in combination. Gemcitabine is a reference chemotherapy in PDAC and can be combined with platinum salts in HCC, making it a potential cytotoxic partner with no expected overlapping toxicities. There is also a rationale for combination with targeted agents such as mTOR or MEK inhibitors, with preclinical data supporting a cooperative relationship between the Ras-ERK and TGF-β pathways[49, 52, 128]. Furthermore, EMT can be a predictive biomarker of response to MEK inhibitors; by attenuating the mesenchymal phenotype of tumour cells, TGF-β inhibition may sensitize them to MEK inhibition[129]. In addition, TGF-β cooperates with hypoxia to induce EMT and VEGF signalling through HIF-1α induction[10, 94, 130]. This provides a rationale in HCC for combination with anti-angiogenic agents such as sorafenib (concomitant or sequential treatment after progression under sorafenib) or with hypoxia-inducing procedures such as arterial embolization. Finally, as TGF-β creates a vicious circle of inflammation and immunosuppression, combination with immunotherapies may also be an option through the restoration of the immune response by TGF-β inhibitors[10, 109]. For example, by restoring lymphocyte cytotoxic activity, TGF-β inhibition may potentiate the effects of anti-CTLA4 or anti-PD1 antibodies.

In conclusion, despite being critical for development and tumour suppression in normal cells, in cancer, alterations of TGF-β pathway signalling do not suppress its signalling but rather change tumour cell fate through intrinsic (SMAD2/3-dependent and -independent pathways rewiring) and extrinsic (microenvironment remodelling) mechanisms. Microenvironment remodelling by TGF-β, in space and time, will generate a more hospitable environment for tumour growth and dissemination. Understanding the mechanisms mediating the dual role of the TGF-β signalling pathway is critical for the development of specific and efficient TGF-β-targeted therapies in PDAC and HCC.

## References

[1] Hidalgo M (2010). Pancreatic cancer. N Engl J Med.

[2] Rhim AD, Mirek ET, Aiello NM, Maitra A, Bailey JM, McAllister F, Reichert M, Beatty GL, Rustgi AK, Vonderheide RH, Leach SD, Stanger BZ (2012). EMT and dissemination precede pancreatic tumor formation. Cell.

[3] Couto OF, Dvorchik I, Carr BI (2007). Causes of death in patients with unresectable hepatocellular carcinoma. Digestive diseases and sciences.

[4] Llovet JM, Ricci S, Mazzaferro V, Hilgard P, Gane E, Blanc JF, de Oliveira AC, Santoro A, Raoul JL, Forner A, Schwartz M, Porta C, Zeuzem S, Bolondi L, Greten TF, Galle PR (2008). Sorafenib in advanced hepatocellular carcinoma. N Engl J Med.

[5] (2012). European Association For The Study Of The L, European Organisation For R and Treatment Of C. EASL-EORTC clinical practice guidelines: management of hepatocellular carcinoma. Journal of hepatology.

[6] Paradis V (2013). Histopathology of hepatocellular carcinoma. Recent results in cancer research Fortschritte der Krebsforschung Progres dans les recherches sur le cancer.

[7] Kitisin K, Saha T, Blake T, Golestaneh N, Deng M, Kim C, Tang Y, Shetty K, Mishra B, Mishra L (2007). Tgf-Beta signaling in development. Science's STKE : signal transduction knowledge environment.

[8] Tian M, Neil JR, Schiemann WP (2011). Transforming growth factor-beta and the hallmarks of cancer. Cellular signalling.

[9] Jakowlew SB (2006). Transforming growth factor-beta in cancer and metastasis. Cancer Metastasis Rev.

[10] Drabsch Y, ten Dijke P (2012). TGF-beta signalling and its role in cancer progression and metastasis. Cancer Metastasis Rev.

[11] Jones S, Zhang X, Parsons DW, Lin JC, Leary RJ, Angenendt P, Mankoo P, Carter H, Kamiyama H, Jimeno A, Hong SM, Fu B, Lin MT, Calhoun ES, Kamiyama M, Walter K (2008). Core signaling pathways in human pancreatic cancers revealed by global genomic analyses. Science.

[12] Ito N, Kawata S, Tamura S, Takaishi K, Shirai Y, Kiso S, Yabuuchi I, Matsuda Y, Nishioka M, Tarui S (1991). Elevated levels of transforming growth factor beta messenger RNA and its polypeptide in human hepatocellular carcinoma. Cancer research.

[13] Shirai Y, Kawata S, Tamura S, Ito N, Tsushima H, Takaishi K, Kiso S, Matsuzawa Y (1994). Plasma transforming growth factor-beta 1 in patients with hepatocellular carcinoma. Comparison with chronic liver diseases. Cancer.

[14] Bedossa P, Peltier E, Terris B, Franco D, Poynard T (1995). Transforming growth factor-beta 1 (TGF-beta 1) and TGF-beta 1 receptors in normal cirrhotic, and neoplastic human livers. Hepatology.

[15] Giannelli G, Mazzocca A, Fransvea E, Lahn M, Antonaci S (2011). Inhibiting TGF-beta signaling in hepatocellular carcinoma. Biochimica et biophysica acta.

[16] Giannelli G, Fransvea E, Marinosci F, Bergamini C, Colucci S, Schiraldi O, Antonaci S (2002). Transforming growth factor-beta1 triggers hepatocellular carcinoma invasiveness via alpha3beta1 integrin. Am J Pathol.

[17] Giannelli G, Bergamini C, Fransvea E, Sgarra C, Antonaci S (2005). Laminin-5 with transforming growth factor-beta1 induces epithelial to mesenchymal transition in hepatocellular carcinoma. Gastroenterology.

[18] Lee D, Chung YH, Kim JA, Lee YS, Lee D, Jang MK, Kim KM, Lim YS, Lee HC, Lee YS (2012). Transforming growth factor beta 1 overexpression is closely related to invasiveness of hepatocellular carcinoma. Oncology.

[19] Ikeguchi M, Iwamoto A, Taniguchi K, Katano K, Hirooka Y (2005). The gene expression level of transforming growth factor-beta (TGF-beta) as a biological prognostic marker of hepatocellular carcinoma. J Exp Clin Cancer Res.

[20] Coppola D, Lu L, Fruehauf JP, Kyshtoobayeva A, Karl RC, Nicosia SV, Yeatman TJ (1998). Analysis of p53, p21WAF1, and TGF-beta1 in human ductal adenocarcinoma of the pancreas: TGF-beta1 protein expression predicts longer survival. American journal of clinical pathology.

[21] Hashimoto K, Nio Y, Sumi S, Toga T, Omori H, Itakura M, Yano S (2001). Correlation between TGF-beta1 and p21 (WAF1/CIP1) expression and prognosis in resectable invasive ductal carcinoma of the pancreas. Pancreas.

[22] Friess H, Yamanaka Y, Buchler M, Ebert M, Beger HG, Gold LI, Korc M (1993). Enhanced expression of transforming growth factor beta isoforms in pancreatic cancer correlates with decreased survival. Gastroenterology.

[23] Culhaci N, Sagol O, Karademir S, Astarcioglu H, Astarcioglu I, Soyturk M, Oztop I, Obuz F (2005). Expression of transforming growth factor-beta-1 and p27Kip1 in pancreatic adenocarcinomas: relation with cell-cycle-associated proteins and clinicopathologic characteristics. BMC Cancer.

[24] Furuta K, Misao S, Takahashi K, Tagaya T, Fukuzawa Y, Ishikawa T, Yoshioka K, Kakumu S (1999). Gene mutation of transforming growth factor beta1 type II receptor in hepatocellular carcinoma. Int J Cancer.

[25] Goggins M, Shekher M, Turnacioglu K, Yeo CJ, Hruban RH, Kern SE (1998). Genetic alterations of the transforming growth factor beta receptor genes in pancreatic and biliary adenocarcinomas. Cancer Res.

[26] Mamiya T, Yamazaki K, Masugi Y, Mori T, Effendi K, Du W, Hibi T, Tanabe M, Ueda M, Takayama T, Sakamoto M (2010). Reduced transforming growth factor-beta receptor II expression in hepatocellular carcinoma correlates with intrahepatic metastasis. Laboratory investigation; a journal of technical methods and pathology.

[27] Wagner M, Kleeff J, Friess H, Buchler MW, Korc M (1999). Enhanced expression of the type II transforming growth factor-beta receptor is associated with decreased survival in human pancreatic cancer. Pancreas.

[28] Murata M, Matsuzaki K, Yoshida K, Sekimoto G, Tahashi Y, Mori S, Uemura Y, Sakaida N, Fujisawa J, Seki T, Kobayashi K, Yokote K, Koike K, Okazaki K (2009). Hepatitis B virus X protein shifts human hepatic transforming growth factor (TGF)-beta signaling from tumor suppression to oncogenesis in early chronic hepatitis B. Hepatology.

[29] Matsuzaki K, Murata M, Yoshida K, Sekimoto G, Uemura Y, Sakaida N, Kaibori M, Kamiyama Y, Nishizawa M, Fujisawa J, Okazaki K, Seki T (2007). Chronic inflammation associated with hepatitis C virus infection perturbs hepatic transforming growth factor signaling promoting cirrhosis and hepatocellular carcinoma. Hepatology.

[30] Brune K, Hong SM, Li A, Yachida S, Abe T, Griffith M, Yang D, Omura N, Eshleman J, Canto M, Schulick R, Klein AP, Hruban RH, Iacobuzio-Donohue C, Goggins M (2008). Genetic and epigenetic alterations of familial pancreatic cancers. Cancer epidemiology, biomarkers & prevention : a publication of the American Association for Cancer Research cosponsored by the American Society of Preventive Oncology.

[31] Hahn SA, Schutte M, Hoque AT, Moskaluk CA, da Costa LT, Rozenblum E, Weinstein CL, Fischer A, Yeo CJ, Hruban RH, Kern SE (1996). DPC4, a candidate tumor suppressor gene at human chromosome 18q21.1. Science.

[32] Neuzillet C, Hammel P, Tijeras-Raballand A, Couvelard A, Raymond E (2013). Targeting the Ras-ERK pathway in pancreatic adenocarcinoma. Cancer Metastasis Rev.

[33] Blackford A, Serrano OK, Wolfgang CL, Parmigiani G, Jones S, Zhang X, Parsons DW, Lin JC, Leary RJ, Eshleman JR, Goggins M, Jaffee EM, Iacobuzio-Donahue CA, Maitra A, Cameron JL, Olino K (2009). SMAD4 gene mutations are associated with poor prognosis in pancreatic cancer. Clinical cancer research : an official journal of the American Association for Cancer Research.

[34] Bachet JB, Marechal R, Demetter P, Bonnetain F, Couvelard A, Svrcek M, Bardier-Dupas A, Hammel P, Sauvanet A, Louvet C, Paye F, Rougier P, Penna C, Vaillant JC, Andre T, Closset J (2012). Contribution of CXCR4 and SMAD4 in predicting disease progression pattern and benefit from adjuvant chemotherapy in resected pancreatic adenocarcinoma. Ann Oncol.

[35] Yao L, Li FJ, Tang ZQ, Gao S, Wu QQ (2012). Smad4 expression in hepatocellular carcinoma differs by hepatitis status. Asian Pacific journal of cancer prevention : APJCP.

[36] Torbenson M, Marinopoulos S, Dang DT, Choti M, Ashfaq R, Maitra A, Boitnott J, Wilentz RE (2002). Smad4 overexpression in hepatocellular carcinoma is strongly associated with transforming growth factor beta II receptor immunolabeling. Human pathology.

[37] Hiwatashi K, Ueno S, Sakoda M, Kubo F, Tateno T, Kurahara H, Mataki Y, Maemura K, Ishigami S, Shinchi H, Natsugoe S (2009). Strong Smad4 expression correlates with poor prognosis after surgery in patients with hepatocellular carcinoma. Ann Surg Oncol.

[38] Wang P, Fan J, Chen Z, Meng ZQ, Luo JM, Lin JH, Zhou ZH, Chen H, Wang K, Xu ZD, Liu LM (2009). Low-level expression of Smad7 correlates with lymph node metastasis and poor prognosis in patients with pancreatic cancer. Ann Surg Oncol.

[39] Xia H, Ooi LL, Hui KM (2013). MicroRNA-216a/217-induced epithelial-mesenchymal transition targets PTEN and SMAD7 to promote drug resistance and recurrence of liver cancer. Hepatology.

[40] Bardeesy N, Cheng KH, Berger JH, Chu GC, Pahler J, Olson P, Hezel AF, Horner J, Lauwers GY, Hanahan D, DePinho RA (2006). Smad4 is dispensable for normal pancreas development yet critical in progression and tumor biology of pancreas cancer. Genes & development.

[41] Wilentz RE, Iacobuzio-Donahue CA, Argani P, McCarthy DM, Parsons JL, Yeo CJ, Kern SE, Hruban RH (2000). Loss of expression of Dpc4 in pancreatic intraepithelial neoplasia: evidence that DPC4 inactivation occurs late in neoplastic progression. Cancer Res.

[42] Morris JPt, Wang SC, Hebrok M (2010). KRAS, Hedgehog, Wnt and the twisted developmental biology of pancreatic ductal adenocarcinoma. Nat Rev Cancer.

[43] Izeradjene K, Combs C, Best M, Gopinathan A, Wagner A, Grady WM, Deng CX, Hruban RH, Adsay NV, Tuveson DA, Hingorani SR (2007). Kras(G12D) and Smad4/Dpc4 haploinsufficiency cooperate to induce mucinous cystic neoplasms and invasive adenocarcinoma of the pancreas. Cancer Cell.

[44] Kojima K, Vickers SM, Adsay NV, Jhala NC, Kim HG, Schoeb TR, Grizzle WE, Klug CA (2007). Inactivation of Smad4 accelerates Kras(G12D)-mediated pancreatic neoplasia. Cancer Res.

[45] Zhang B, Halder SK, Kashikar ND, Cho YJ, Datta A, Gorden DL, Datta PK (2010). Antimetastatic role of Smad4 signaling in colorectal cancer. Gastroenterology.

[46] Coulouarn C, Factor VM, Thorgeirsson SS (2008). Transforming growth factor-beta gene expression signature in mouse hepatocytes predicts clinical outcome in human cancer. Hepatology.

[47] Mu X, Lin S, Yang J, Chen C, Chen Y, Herzig MC, Washburn K, Halff GA, Walter CA, Sun B, Sun LZ (2013). TGF-beta signaling is often attenuated during, Hepatotumorigenesis but is retained for the malignancy of hepatocellular carcinoma cells. PLoS One.

[48] Dooley S, ten Dijke P (2012). TGF-beta in progression of liver disease. Cell and tissue research.

[49] Matsuzaki K (2011). Smad phosphoisoform signaling specificity: the right place at the right time. Carcinogenesis.

[50] Wu K, Ding J, Chen C, Sun W, Ning BF, Wen W, Huang L, Han T, Yang W, Wang C, Li Z, Wu MC, Feng GS, Xie WF, Wang HY (2012). Hepatic transforming growth factor beta gives rise to tumor-initiating cells and promotes liver cancer development. Hepatology.

[51] Mazzocca A, Fransvea E, Dituri F, Lupo L, Antonaci S, Giannelli G (2010). Down-regulation of connective tissue growth factor by inhibition of transforming growth factor beta blocks the tumor-stroma cross-talk and tumor progression in hepatocellular carcinoma. Hepatology.

[52] Cano CE, Motoo Y, Iovanna JL (2010). Epithelial-to-mesenchymal transition in pancreatic adenocarcinoma. ScientificWorldJournal.

[53] Binker MG, Binker-Cosen AA, Gaisano HY, de Cosen RH, Cosen-Binker LI (2011). TGF-beta1 increases invasiveness of SW1990 cells through Rac1/ROS/NF-kappaB/IL-6/MMP-2. Biochemical and biophysical research communications.

[54] Ottaviano AJ, Sun L, Ananthanarayanan V, Munshi HG (2006). Extracellular matrix-mediated membrane-type 1 matrix metalloproteinase expression in pancreatic ductal cells is regulated by transforming growth factor-beta1. Cancer research.

[55] Ellenrieder V, Hendler SF, Ruhland C, Boeck W, Adler G, Gress TM (2001). TGF-beta-induced invasiveness of pancreatic cancer cells is mediated by matrix metalloproteinase-2 and the urokinase plasminogen activator system. Int J Cancer.

[56] Fransvea E, Mazzocca A, Antonaci S, Giannelli G (2009). Targeting transforming growth factor (TGF)-betaRI inhibits activation of beta1 integrin and blocks vascular invasion in hepatocellular carcinoma. Hepatology.

[57] Fransvea E, Angelotti U, Antonaci S, Giannelli G (2008). Blocking transforming growth factor-beta up-regulates E-cadherin and reduces migration and invasion of hepatocellular carcinoma cells. Hepatology.

[58] Mima K, Hayashi H, Kuroki H, Nakagawa S, Okabe H, Chikamoto A, Watanabe M, Beppu T, Baba H (2013). Epithelial-mesenchymal transition expression profiles as a prognostic factor for disease-free survival in hepatocellular carcinoma: Clinical significance of transforming growth factor-beta signaling. Oncology letters.

[59] Hezel AF, Deshpande V, Zimmerman SM, Contino G, Alagesan B, O'Dell MR, Rivera LB, Harper J, Lonning S, Brekken RA, Bardeesy N (2012). TGF-beta and alphavbeta6 integrin act in a common pathway to suppress pancreatic cancer progression. Cancer Res.

[60] Mu Y, Gudey SK, Landstrom M (2012). Non-Smad signaling pathways. Cell and tissue research.

[61] Hoshida Y, Nijman SM, Kobayashi M, Chan JA, Brunet JP, Chiang DY, Villanueva A, Newell P, Ikeda K, Hashimoto M, Watanabe G, Gabriel S, Friedman SL, Kumada H, Llovet JM, Golub TR (2009). Integrative transcriptome analysis reveals common molecular subclasses of human hepatocellular carcinoma. Cancer research.

[62] Lachenmayer A, Alsinet C, Savic R, Cabellos L, Toffanin S, Hoshida Y, Villanueva A, Minguez B, Newell P, Tsai HW, Barretina J, Thung S, Ward SC, Bruix J, Mazzaferro V, Schwartz M (2012). Wnt-pathway activation in two molecular classes of hepatocellular carcinoma and experimental modulation by sorafenib. Clinical cancer research : an official journal of the American Association for Cancer Research.

[63] Romero D, Iglesias M, Vary CP, Quintanilla M (2008). Functional blockade of Smad4 leads to a decrease in beta-catenin levels and signaling activity in human pancreatic carcinoma cells. Carcinogenesis.

[64] Erkan M, Hausmann S, Michalski CW, Fingerle AA, Dobritz M, Kleeff J, Friess H (2012). The role of stroma in pancreatic cancer: diagnostic and therapeutic implications. Nature reviews Gastroenterology & hepatology.

[65] Neesse A, Michl P, Frese KK, Feig C, Cook N, Jacobetz MA, Lolkema MP, Buchholz M, Olive KP, Gress TM, Tuveson DA (2011). Stromal biology and therapy in pancreatic cancer. Gut.

[66] Couvelard A, O'Toole D, Leek R, Turley H, Sauvanet A, Degott C, Ruszniewski P, Belghiti J, Harris AL, Gatter K, Pezzella F (2005). Expression of hypoxia-inducible factors is correlated with the presence of a fibrotic focus and angiogenesis in pancreatic ductal adenocarcinomas. Histopathology.

[67] Watanabe I, Hasebe T, Sasaki S, Konishi M, Inoue K, Nakagohri T, Oda T, Mukai K, Kinoshita T (2003). Advanced pancreatic ductal cancer: fibrotic focus and beta-catenin expression correlate with outcome. Pancreas.

[68] Luo G, Long J, Zhang B, Liu C, Xu J, Ni Q, Yu X (2012). Stroma and pancreatic ductal adenocarcinoma: an interaction loop. Biochim Biophys Acta.

[69] Erkan M, Michalski CW, Rieder S, Reiser-Erkan C, Abiatari I, Kolb A, Giese NA, Esposito I, Friess H, Kleeff J (2008). The activated stroma index is a novel and independent prognostic marker in pancreatic ductal adenocarcinoma. Clin Gastroenterol Hepatol.

[70] Apte MV, Park S, Phillips PA, Santucci N, Goldstein D, Kumar RK, Ramm GA, Buchler M, Friess H, McCarroll JA, Keogh G, Merrett N, Pirola R, Wilson JS (2004). Desmoplastic reaction in pancreatic cancer: role of pancreatic stellate cells. Pancreas.

[71] Lohr M, Schmidt C, Ringel J, Kluth M, Muller P, Nizze H, Jesnowski R (2001). Transforming growth factor-beta1 induces desmoplasia in an experimental model of human pancreatic carcinoma. Cancer research.

[72] Bachem MG, Schunemann M, Ramadani M, Siech M, Beger H, Buck A, Zhou S, Schmid-Kotsas A, Adler G (2005). Pancreatic carcinoma cells induce fibrosis by stimulating proliferation and matrix synthesis of stellate cells. Gastroenterology.

[73] Vonlaufen A, Joshi S, Qu C, Phillips PA, Xu Z, Parker NR, Toi CS, Pirola RC, Wilson JS, Goldstein D, Apte MV (2008). Pancreatic stellate cells: partners in crime with pancreatic cancer cells. Cancer research.

[74] Hwang RF, Moore TT, Hattersley MM, Scarpitti M, Yang B, Devereaux E, Ramachandran V, Arumugam T, Ji B, Logsdon CD, Brown JL, Godin R (2012). Inhibition of the hedgehog pathway targets the tumor-associated stroma in pancreatic cancer. Molecular cancer research : MCR.

[75] Spector I, Zilberstein Y, Lavy A, Nagler A, Genin O, Pines M (2012). Involvement of host stroma cells and tissue fibrosis in pancreatic tumor development in transgenic mice. PLoS One.

[76] Xu Z, Vonlaufen A, Phillips PA, Fiala-Beer E, Zhang X, Yang L, Biankin AV, Goldstein D, Pirola RC, Wilson JS, Apte MV (2010). Role of pancreatic stellate cells in pancreatic cancer metastasis. Am J Pathol.

[77] Apte MV, Wilson JS, Lugea A, Pandol SJ (2013). A starring role for stellate cells in the pancreatic cancer microenvironment. Gastroenterology.

[78] Apte MV, Wilson JS (2012). Dangerous liaisons: pancreatic stellate cells and pancreatic cancer cells. Journal of gastroenterology and hepatology.

[79] Provenzano PP, Hingorani SR (2013). Hyaluronan, fluid pressure, and stromal resistance in pancreas cancer. Br J Cancer.

[80] Erkan M, Reiser-Erkan C, Michalski CW, Deucker S, Sauliunaite D, Streit S, Esposito I, Friess H, Kleeff J (2009). Cancer-stellate cell interactions perpetuate the hypoxia-fibrosis cycle in pancreatic ductal adenocarcinoma. Neoplasia.

[81] Masamune A, Kikuta K, Watanabe T, Satoh K, Hirota M, Shimosegawa T (2008). Hypoxia stimulates pancreatic stellate cells to induce fibrosis and angiogenesis in pancreatic cancer. American journal of physiology Gastrointestinal and liver physiology.

[82] Eguchi D, Ikenaga N, Ohuchida K, Kozono S, Cui L, Fujiwara K, Fujino M, Ohtsuka T, Mizumoto K, Tanaka M (2013). Hypoxia enhances the interaction between pancreatic stellate cells and cancer cells via increased secretion of connective tissue growth factor. The Journal of surgical research.

[83] Bergers G, Hanahan D (2008). Modes of resistance to anti-angiogenic therapy. Nat Rev Cancer.

[84] Kindler HL, Niedzwiecki D, Hollis D, Sutherland S, Schrag D, Hurwitz H, Innocenti F, Mulcahy MF, O'Reilly E, Wozniak TF, Picus J, Bhargava P, Mayer RJ, Schilsky RL, Goldberg RM (2010). Gemcitabine plus bevacizumab compared with gemcitabine plus placebo in patients with advanced pancreatic cancer: phase III trial of the Cancer and Leukemia Group B (CALGB 80303). Journal of clinical oncology : official journal of the American Society of Clinical Oncology.

[85] Erkan M (2013). The role of pancreatic stellate cells in pancreatic cancer. Pancreatology.

[86] Casanovas O, Hicklin DJ, Bergers G, Hanahan D (2005). Drug resistance by evasion of antiangiogenic targeting of VEGF signaling in late-stage pancreatic islet tumors. Cancer Cell.

[87] Paez-Ribes M, Allen E, Hudock J, Takeda T, Okuyama H, Vinals F, Inoue M, Bergers G, Hanahan D, Casanovas O (2009). Antiangiogenic therapy elicits malignant progression of tumors to increased local invasion and distant metastasis. Cancer Cell.

[88] Pennacchietti S, Michieli P, Galluzzo M, Mazzone M, Giordano S, Comoglio PM (2003). Hypoxia promotes invasive growth by transcriptional activation of the met protooncogene. Cancer cell.

[89] Kitajima Y, Ide T, Ohtsuka T, Miyazaki K (2008). Induction of hepatocyte growth factor activator gene expression under hypoxia activates the hepatocyte growth factor/c-Met system via hypoxia inducible factor-1 in pancreatic cancer. Cancer science.

[90] Schioppa T, Uranchimeg B, Saccani A, Biswas SK, Doni A, Rapisarda A, Bernasconi S, Saccani S, Nebuloni M, Vago L, Mantovani A, Melillo G, Sica A (2003). Regulation of the chemokine receptor CXCR4 by hypoxia. The Journal of experimental medicine.

[91] Staller P, Sulitkova J, Lisztwan J, Moch H, Oakeley EJ, Krek W (2003). Chemokine receptor CXCR4 downregulated by von Hippel-Lindau tumour suppressor pVHL. Nature.

[92] Hashimoto O, Shimizu K, Semba S, Chiba S, Ku Y, Yokozaki H, Hori Y (2011). Hypoxia induces tumor aggressiveness and the expansion of CD133-positive cells in a hypoxia-inducible factor-1alpha-dependent manner in pancreatic cancer cells Pathobiology. journal of immunopathology molecular and cellular biology.

[93] Guillaumond F, Leca J, Olivares O, Lavaut MN, Vidal N, Berthezene P, Dusetti NJ, Loncle C, Calvo E, Turrini O, Iovanna JL, Tomasini R, Vasseur S (2013). Strengthened glycolysis under hypoxia supports tumor symbiosis and hexosamine biosynthesis in pancreatic adenocarcinoma. Proceedings of the National Academy of Sciences of the United States of America.

[94] Mimeault M, Batra SK (2013). Hypoxia-inducing factors as master regulators of stemness properties and altered metabolism of cancer- and metastasis-initiating cells. J Cell Mol Med.

[95] Melisi D, Ishiyama S, Sclabas GM, Fleming JB, Xia Q, Tortora G, Abbruzzese JL, Chiao PJ (2008). LY2109761, a novel transforming growth factor beta receptor type I and type II dual inhibitor, as a therapeutic approach to suppressing pancreatic cancer metastasis. Molecular cancer therapeutics.

[96] Gaspar NJ, Li L, Kapoun AM, Medicherla S, Reddy M, Li G, O'Young G, Quon D, Henson M, Damm DL, Muiru GT, Murphy A, Higgins LS, Chakravarty S, Wong DH (2007). Inhibition of transforming growth factor beta signaling reduces pancreatic adenocarcinoma growth and invasiveness. Molecular pharmacology.

[97] Schlingensiepen KH, Jaschinski F, Lang SA, Moser C, Geissler EK, Schlitt HJ, Kielmanowicz M, Schneider A (2011). Transforming growth factor-beta 2 gene silencing with trabedersen (AP 12009) in pancreatic cancer. Cancer science.

[98] (2012). European Association for Study of L, European Organisation for R and Treatment of C. EASL-EORTC clinical practice guidelines: management of hepatocellular carcinoma. European journal of cancer.

[99] Frampas E, Lassau N, Zappa M, Vullierme MP, Koscielny S, Vilgrain V (2013). Advanced Hepatocellular Carcinoma: early evaluation of response to targeted therapy and prognostic value of Perfusion CT and Dynamic Contrast Enhanced-Ultrasound. Preliminary results. European journal of radiology.

[100] (2009). International Consensus Group for Hepatocellular NeoplasiaThe International Consensus Group for Hepatocellular N. Pathologic diagnosis of early hepatocellular carcinoma: a report of the international consensus group for hepatocellular neoplasia. Hepatology.

[101] Messerini L, Novelli L, Comin CE (2004). Microvessel density and clinicopathological characteristics in hepatitis C virus and hepatitis B virus related hepatocellular carcinoma. Journal of clinical pathology.

[102] Harmon CS, DePrimo SE, Raymond E, Cheng AL, Boucher E, Douillard JY, Lim HY, Kim JS, Lechuga MJ, Lanzalone S, Lin X, Faivre S (2011). Mechanism-related circulating proteins as biomarkers for clinical outcome in patients with unresectable hepatocellular carcinoma receiving sunitinib. Journal of translational medicine.

[103] ten Dijke P, Arthur HM (2007). Extracellular control of TGFbeta signalling in vascular development and disease. Nature reviews Molecular cell biology.

[104] Ito N, Kawata S, Tamura S, Shirai Y, Kiso S, Tsushima H, Matsuzawa Y (1995). Positive correlation of plasma transforming growth factor-beta 1 levels with tumor vascularity in hepatocellular carcinoma. Cancer letters.

[105] Sanchez-Elsner T, Botella LM, Velasco B, Corbi A, Attisano L, Bernabeu C (2001). Synergistic cooperation between hypoxia and transforming growth factor-beta pathways on human vascular endothelial growth factor gene expression. The Journal of biological chemistry.

[106] Mazzocca A, Fransvea E, Lavezzari G, Antonaci S, Giannelli G (2009). Inhibition of transforming growth factor beta receptor I kinase blocks hepatocellular carcinoma growth through neo-angiogenesis regulation. Hepatology.

[107] Murawaki Y, Ikuta Y, Nishimura Y, Koda M, Kawasaki H (1996). Serum markers for fbrosis and plasma transforming growth factor-beta 1 in patients with hepatocellular carcinoma in comparison with patients with liver cirrhosis. Journal of gastroenterology and hepatology.

[108] Wojtowicz-Praga S (2003). Reversal of tumor-induced immunosuppression by TGF-beta inhibitors. Invest New Drugs.

[109] Yang L (2010). TGFbeta and cancer metastasis: an infammation link. Cancer Metastasis Rev.

[110] Kulkarni AB, Ward JM, Yaswen L, Mackall CL, Bauer SR, Huh CG, Gress RE, Karlsson S (1995). Transforming growth factor-beta 1 null mice. An animal model for inflammatory disorders. Am J Pathol.

[111] Shull MM, Ormsby I, Kier AB, Pawlowski S, Diebold RJ, Yin M, Allen R, Sidman C, Proetzel G, Calvin D (1992). Targeted disruption of the mouse transforming growth factor-beta 1 gene results in multifocal inflammatory disease. Nature.

[112] von Bernstorff W, Voss M, Freichel S, Schmid A, Vogel I, Johnk C, Henne-Bruns D, Kremer B, Kalthoff H (2001). Systemic and local immunosuppression in pancreatic cancer patients. Clin Cancer Res.

[113] Truty MJ, Urrutia R (2007). Basics of TGF-beta and pancreatic cancer. Pancreatology.

[114] Achyut BR, Yang L (2011). Transforming growth factor-beta in the gastrointestinal and hepatic tumor microenvironment. Gastroenterology.

[115] Yang L, Pang Y, Moses HL (2010). TGF-beta and immune cells: an important regulatory axis in the tumor microenvironment and progression. Trends in immunology.

[116] Helmut Oettle TS, Thomas Luger, Roland M. Schmid, Goetz von Wichert, Esther Endlicher, Claus Garbe, Katharina K. Kaehler, Alexander Enk, Anneliese Schneider, Tanja Rothhammer-Hampl, Susanne Grosser, Peter Kiessling (2012). Final results of a phase I/II study in patients with pancreatic cancer, malignant, melanoma and colorectal carcinoma with trabedersen. ASCO: Journal of clinical oncology.

[117] Nemunaitis J, Dillman RO, Schwarzenberger PO, Senzer N, Cunningham C, Cutler J, Tong A, Kumar P, Pappen B, Hamilton C, DeVol E, Maples PB, Liu L, Chamberlin T, Shawler DL, Fakhrai H (2006). Phase II study of belagenpumatucel-L, a transforming growth factor beta-2 antisense gene-modifed allogeneic tumor cell vaccine in non-small-cell lung cancer. Journal of clinical oncology : official journal of the American Society of Clinical Oncology.

[118] Zubeldia IG, Bleau AM, Redrado M, Serrano D, Agliano A, Gil-Puig C, Vidal-Vanaclocha F, Lecanda J, Calvo A (2013). Epithelial to mesenchymal transition and cancer stem cell phenotypes leading to liver metastasis are abrogated by the novel TGFbeta1-targeting peptides P17 and P144. Experimental cell research.

[119] Dituri F, Mazzocca A, Peidro FJ, Papappicco P, Fabregat I, De Santis F, Paradiso A, Sabba C, Giannelli G (2013). Differential Inhibition of the TGF-beta Signaling Pathway in HCC Cells Using the Small Molecule Inhibitor LY2157299 and the D10 Monoclonal Antibody against TGF-beta Receptor Type II. PloS one.

[120] Bhola NE, Balko JM, Dugger TC, Kuba MG, Sanchez V, Sanders M, Stanford J, Cook RS, Arteaga CL (2013). TGF-beta inhibition enhances chemotherapy action against triple-negative breast cancer. The Journal of clinical investigation.

[121] Sandrine J. Faivre AS, Robin Katie Kelley, Philippe Merle, Ed Gane, Jean-Yves Douillard, Dirk Waldschmidt, Mary Frances Mulcahy, Charlotte Costentin, Beatriz Minguez, Pasqua Papappicco, Ivelina Gueorguieva, Ann Cleverly, Durisala Desaiah, Michael M. F. Lahn, Nicola Murray, Karim A. Benhadji, Eric Raymond, Gianluigi Giannelli (2013). Randomized dose comparison phase II study of the oral transforming growth factor-beta (TGF-ß) receptor I kinase inhibitor LY2157299 monohydrate (LY) in patients with advanced hepatocellular carcinoma (HCC). Journal of clinical oncology.

[122] Cukierman E, Pankov R, Stevens DR, Yamada KM (2001). Taking cell-matrix adhesions to the third dimension. Science.

[123] Yamada KM, Cukierman E (2007). Modeling tissue morphogenesis and cancer in 3D. Cell.

[124] Godoy P, Hengstler JG, Ilkavets I, Meyer C, Bachmann A, Muller A, Tuschl G, Mueller SO, Dooley S (2009). Extracellular matrix modulates sensitivity of hepatocytes to fibroblastoid dedifferentiation and transforming growth factor beta-induced apoptosis. Hepatology.

[125] Sempere LF, Gunn JR, Korc M (2011). A novel 3-dimensional culture system uncovers growth stimulatory actions by TGFbeta in pancreatic cancer cells. Cancer Biol Ther.

[126] Froeling FE, Marshall JF, Kocher HM (2010). Pancreatic cancer organotypic cultures. Journal of biotechnology.

[127] Biswas S, Guix M, Rinehart C, Dugger TC, Chytil A, Moses HL, Freeman ML, Arteaga CL (2007). Inhibition of TGF-beta with neutralizing antibodies prevents radiation-induced acceleration of metastatic cancer progression. J Clin Invest.

[128] Gadir N, Jackson DN, Lee E, Foster DA (2008). Defective TGF-beta signaling sensitizes human cancer cells to rapamycin. Oncogene.

[129] Jing J, Greshock J, Holbrook JD, Gilmartin A, Zhang X, McNeil E, Conway T, Moy C, Laquerre S, Bachman K, Wooster R, Degenhardt Y (2012). Comprehensive predictive biomarker analysis for MEK inhibitor GSK1120212. Mol Cancer Ther.

[130] Copple BL (2010). Hypoxia stimulates hepatocyte epithelial to mesenchymal transition by hypoxia-inducible factor and transforming growth factor-beta-dependent mechanisms. Liver international : official journal of the International Association for the Study of the Liver.

[131] Apte MV, Haber PS, Applegate TL, Norton ID, McCaughan GW, Korsten MA, Pirola RC, Wilson JS (1998). Periacinar stellate shaped cells in rat pancreas: identification, isolation, and culture. Gut.

[132] Duner S, Lopatko Lindman J, Ansari D, Gundewar C, Andersson R (2010). Pancreatic cancer: the role of pancreatic stellate cells in tumor progression. Pancreatology.

[133] Jaster R (2004). Molecular regulation of pancreatic stellate cell function. Mol Cancer.

[134] Atzori L, Poli G, Perra A (2009). Hepatic stellate cell: a star cell in the liver. The international journal of biochemistry & cell biology.

[135] Ohnishi H, Miyata T, Yasuda H, Satoh Y, Hanatsuka K, Kita H, Ohashi A, Tamada K, Makita N, Iiri T, Ueda N, Mashima H, Sugano K (2004). Distinct roles of Smad2-, Smad3-, and ERK-dependent pathways in transforming growth factor-beta1 regulation of pancreatic stellate cellular functions. J Biol Chem.

[136] Kang N, Gores GJ, Shah VH (2011). Hepatic stellate cells: partners in crime for liver metastases. Hepatology.

[137] Vonlaufen A, Phillips PA, Xu Z, Goldstein D, Pirola RC, Wilson JS, Apte MV (2008). Pancreatic stellate cells and pancreatic cancer cells: an unholy alliance. Cancer research.

[138] Derynck R, Jarrett JA, Chen EY, Eaton DH, Bell JR, Assoian RK, Roberts AB, Sporn MB, Goeddel DV (1985). Human transforming growth factor-beta complementary DNA sequence and expression in normal and transformed cells. Nature.

[139] Massague J, Wotton D (2000). Transcriptional control by the TGF-beta/Smad signaling system. The EMBO journal.

[140] Ross S, Hill CS (2008). How the Smads regulate transcription. The international journal of biochemistry & cell biology.

[141] Inman GJ, Nicolas FJ, Hill CS (2002). Nucleocytoplasmic shuttling of Smads 2, 3, and 4 permits sensing of TGF-beta receptor activity. Molecular cell.

[142] Derynck R, Zhang YE (2003). Smad-dependent and Smad-independent pathways in TGF-beta family signalling. Nature.

[143] Bierie B, Moses HL (2006). Tumour microenvironment: TGFbeta: the molecular Jekyll and Hyde of cancer. Nature reviews Cancer.

[144] Yingling JM, Blanchard KL, Sawyer JS (2004). Development of TGF-beta signalling inhibitors for cancer therapy. Nature reviews Drug discovery.

